# The pathological features and prognoses of intraductal papillary mucinous neoplasm and mucinous cystic neoplasm after surgical resection: a single institution series

**DOI:** 10.1186/s12957-020-02063-8

**Published:** 2020-11-04

**Authors:** Yuqiong Li, Zhongfei Zhu, Lisi Peng, Zhendong Jin, Liqi Sun, Bin Song

**Affiliations:** 1 Department of Gastroenterology, Changhai Hospital, Navy Medical University, 168 Changhai Road, Shanghai, 200433 China; 2 Department of Hepatobiliary Pancreatic Surgery, Changhai Hospital, Navy Medical University, 168 Changhai Road, Shanghai, 200433 China

**Keywords:** Intraductal papillary mucinous neoplasm (IPMN), Mucinous cystic neoplasm (MCN), Pathological features, Prognosis, Recurrence

## Abstract

**Background:**

Intraductal papillary mucinous neoplasms (IPMNs) and mucinous cystic neoplasms (MCNs) represent the tumors with malignant transformation potential. The objective of the study was to verify their pathological characteristics, prognoses, and recurrence factors.

**Methods:**

Two hundred eighteen IPMNs and 27 MCNs resected at a single institution were included. The demographic, preoperative, histopathological, and follow-up data of the patients were recorded and analyzed. Overall survival (OS) and disease-free survival (DFS) were defined as the interval from the date of initial surgery to death or the last follow-up (OS) and to diagnosis of recurrence or death at follow-up (DFS).

**Results:**

Of the 218 IPMN and 27 MCN patients, 93 (42.7%) and 8 (29.6%) cases were malignant, respectively. IPMNs occurred in older patients compared with MCN patients (median 63 years vs 54 years, *P* < 0.0001), and MCNs occurred exclusively in females (100%). Of the overall study cohort, the pathological specimens presented peripheral invasion in 37 (15.1%) patients and incisal margin invasion was observed in 46 (18.8%) patients. After a median follow-up of 34 months, 37 (14.9%) patients relapsed. The 5-year OS and DFS rates of IPMNs were 97.5% and 80.6%; and the OS and DFS rates of MCNs were 95.7% and 87.0%, respectively. There were four independent risk factors associated with recurrence: pathological diagnoses with malignancy (odds ratio, OR = 3.65), presence of oncocytic type for IPMN (OR = 1.69), peripheral invasion (OR = 12.87), and incisal margin invasion (OR = 1.99).

**Conclusions:**

IPMNs and MCNs are indolent tumors with favorable prognoses after surgical resection in terms of their relatively high OS and DFS rate. Patients with malignant pathological-related diagnoses should accept strict tumor surveillance in view of their higher risk of recurrence.

## Introduction

Cystic neoplasms of the pancreas (PCNs) have a wide clinicopathologic spectrum. Their prevalence ranges from 1.9 to 49.1% in different races [1–3]. More than half of them are intraductal papillary mucinous neoplasms (IPMNs) [4–6], and 10–45% are mucinous cystic neoplasms (MCNs) [7, 8]. IPMNs and MCNs represent tumors that have the potential to progress into invasive cancer. They have been classified separately by the World Health Organization since 1996 [9]. However, it was not until 2006 that the diagnostic criteria and terminology of MCN and branch-duct (BD) IPMN were clearly elucidated, and ovarian-type stroma was recognized as a character of MCN [10].

Except for the distinctive ovarian-type stroma MCNs, other types of MCNs generally do not communicate with the pancreatic ductal system and occur almost exclusively in the body and tail regions of the pancreas. MCNs have a tendency to be quite large with a median size of 4–5 cm. MCNs have a strong female preponderance (20:1) and are usually diagnosed in the middle age (fourth and fifth decades) of life [11]. However, IPMNs are generally defined as cystic tumors involving the ductal system. They affect male and female equally between the elder age (sixth and seventh decades) of life and affect the head of the pancreas more often than any other regions of the pancreas [12].

In other words, IPMNs and MCNs are two different pathological types of tumors with different biological behaviors, although they both have a neoplastic mucin-producing epithelial lining. Due to the difficulty of distinguishing IPMN from MCN by preoperative imaging modalities, many guidelines recommend MCN and IPMN as the same strategies [10, 13]. The core strategy for the management of MCNs and IPMNs is surgical resection of tumors with imaging high-risk features [14, 15]. However, few literatures focused on the differences in postoperative clinicopathologic features, recurrence rate, survival rate, and recurrence factors between MCNs and IPMNs. As a result, we may be unable to know whether the same postoperative surveillance strategy is appropriate for them.

The goal of the study is to clarify the clinicopathologic features and long-term outcomes following surgical resections of MCNs and IPMNs in a large single-institution, pathology confirmed series to date. Their risk factors of postoperative recurrence were also evaluated.

## Methods

### Study population

We conducted a retrospective study at Changhai Hospital affiliated with Navy/second Medical University, one of the largest pancreatic disease research centers in Shanghai, China. The Institutional Review Board of Changhai Hospital approved the study. Patients who underwent surgical resection from January 2013 to June 2019 for an identified pancreatic cyst and whose final pathological outcomes were identified with MCNs and IPMNs were included in our study. January 2013 was chosen as the start time because the 2012 International Consensus Guidelines influenced our clinical strategy [11]. All the patients underwent contrast-enhanced computed tomography (CT) and/or magnetic resonance imaging (MRI) preoperatively. Endoscopic ultrasound (EUS) was performed in some patients. And at least 12 months of postoperative imaging follow-up was required for the included patients. Exclusion criteria included (1) cases with an undefined grade of dysplasia, (2) specimens obtained from re-resections, (3) concomitant other neoplasms on final pathology (e.g., neuroendocrine tumor, cholangiocarcinoma), (4) patients with a postoperative follow-up period < 12 months, and (5) patients with unavailable imaging or pathological data.

The decision on surgical treatment was made by our multidisciplinary hepato-pancreatobiliary team for the vast majority of patients in accordance with the International Consensus Guideline [10, 11]. The additional surgical indications were high carcinoembryonic antigen (CEA) level and/or positive cytology in cyst fluid obtained by EUS-guided fine needle aspiration (FNA) or endoscopic retrograde pancreatography. The choices of surgical procedures depended on the location, degree, and extent of diseases.

Patient demographics, clinical history, surgical procedure, detailed pathologic characteristics, margin status, peripheral tissue invasion status, progression details, and follow-up details were extracted from the electronic medical records.

### Histopathological assessment

Surgically resected specimens were reviewed under the supervision of at least one senior pathologist specializing in pancreatic pathology.

The surgical procedures consisted of distal pancreatectomy (DP), pancreaticoduodenectomy (PD), total pancreatectomy (TP), central pancreatectomy (CP), and enucleation. The pathologic finding of IPMNs and MCNs were graded as low grade of dysplasia (LGD), intermediate grade of dysplasia (IGD), high grade of dysplasia (HGD), or invasive cancer based on the highest level of dysplasia encountered in the specimens [16]. The patients who had HGD or invasive cancer were considered as malignant, and the patients with other grades of dysplasia were considered as non-invasive. The duct subtypes of IPMNs are main duct (MD) IPMN, BD-IPMN, and mixed type (MT) of IPMN. The epithelial subtypes of IPMNs are gastric, intestinal, pancreatobiliary, oncocytic, and mixed type. The margin status was defined as negative, non-invasive component (LGD, IGD, PanIN, and invasive cancer > 5 mm from the incisal margin), or invasive component (invasive cancer ≤ 5 mm from the incisal margin). Peripheral tissue invasion status was consisted of perineural invasion, vascular invasion, cancerization of ducts, lymphatic metastasis, common bile duct invasion, peripancreatic fat invasion, and duodenum invasion.

### Definition of recurrence

Patients were examined every 6–12 months after surgery by serum tumor markers and imaging examinations such as CT, MRI, and EUS.

Recurrence was defined as a tumor confirmed by radiology or histology during postoperative follow-up. The sites of recurrence were classified as the residual pancreas (locoregional) and outside the pancreas (extra-pancreatic). The locoregional recurrence was defined as metachronous development of a tumor in the residual pancreas. Extra-pancreatic recurrence was defined as appearance of tumors outside the pancreas, including the peripancreatic areas (local lymph nodes, nerve plexus), lungs, liver, and peritoneal cavity.

### Statistical analysis

The clinical features, histopathological outcomes, and recurrence data were compared between the two groups (IPMNs group and MCNs group). Categorical data were expressed as frequencies (%) with total observations (*n*), and the chi-square test or Fisher’s exact test was used for comparison between groups. Continuous data were summarized as medians with interquartile ranges (IQR) and were compared between groups using the Student’s *t* or Mann-Whitney *U* tests.

Overall survival (OS) was defined as the interval from the date of initial surgery to death or the last follow-up. Perioperative deaths were excluded. Disease-free survival (DFS) was defined as the interval from the date of initial surgery to diagnosis of recurrence or death at follow-up. OS and DFS were assessed by the Kaplan-Meier method with log-rank comparisons. A logistic regression model was established to determine the pathological factors associated with recurrence. All statistical analyses were performed with the SPSS software (version 20; IBM Corp, Somers, NY). Statistical significance was set at *P* values < 0.05.

## Results

### Demographics and clinicopathological features

From January 2013 to June 2019, 245 patients with pathologically diagnosed IPMNs and MCNs were included in this study. Of the 245 patients, 218 (89%) were IPMNs and 27 (11%) were MCNs. Of 218 IPMNs, 125 (57.3%) were non-invasive, 93 (42.7%) were malignant, and 148 (67.9%) of them were males. Of 27 MCNs, 19 (70.4%) were non-invasive, 8 (29.6%) were malignant, and all of them were females. Detailed patient demographics and clinicopathological features are presented in Table 1.

**Table 1:** Demographic and clinicopathological features of the study cohort

Demographics	IPMN				MCN				IPMN vs MCN
	All	Noninvasive	Malignant	*p* value^a^	All	Noninvasive	Malignant	*p* value^b^	*p* value^c^
Total subjects, (*n*)	218	125	93		27	19	8		
Age, median (IQR)	63.0 (56.0–70.0)	63.0 (55.5–70.0)	64.0 (56.5–71.0)	0.45	54.0 (41.0–60.0)	53.0 (35.0–57.0)	57.0 (49.0–71.3)	0.12	**< 0.0001**
Female, % (*n*)	32.1 (70)	30.4 (38)	34.4 (32)	0.53	100 (27)	100 (19)	100 (8)	< 0.0001	**< 0.0001**
Diabetes mellitus, % (*n*)	24.3 (53)	15.2 (19)	36.6 (34)	**< 0.0001**	18.5 (5)	15.8 (3)	25 (2)	0.62	0.5
**Surgical resections and pathological outcomes**									
**Resection procedure, % (*n*)**									
TP	6.9 (15)	4.8 (6)	9 (9.7)	0.16	0 (0)	0 (0)	0 (0)	-	0.39
PD	64.2 (140)	58.4 (73)	69.1 (67)	**0.04**	14.8 (4)	10.2 (2)	25 (2)	0.56	**< 0.0001**
DP	19.3 (42)	25.6 (32)	10.8 (10)	**0.006**	81.5 (22)	89.8 (17)	63 (5)	0.14	**< 0.0001**
CP	7.8 (17)	8.0 (10)	7.5 (7)	0.9	0 (0)	0 (0)	0 (0)	-	0.23
Enucleation	1.8 (4)	3.2 (4)	0 (0)	0.14	3.7 (1)	0 (0)	13 (1)	0.32	0.45
Tumor size (mm), median (IQR)	40(23.8-70)	38 (21.5–75)	40 (27.5–67.5)	0.89	45 (35–65)	40 (35–55)	58.5 (35–68.8)	0.39	0.89
Morphological type for IPMN (BD/MD/MT), *n*	34/80/104	21/59/45	13/21/59	0.57					
Epithelial subtype for IPMN (G/I/P/O/M), *n*	91/81/26/9/11	54/47/8/2/4	27/34/18/7/7	**0.006**					
**Peripheral invasion, %(*n*)**									
Perineural invasion	12.8 (28)	0 (0)	30.1 (28)		14.8 (4)	0 (0)	50.0 (4)		1.0
Vascular invasion	8.3 (18)	0 (0)	19.4 (18)		0 (0)	0 (0)	0 (0)		0.23
Cancerization of ducts	5.5 (12)	0 (0)	12.9 (12)		7.4 (2)	0 (0)	25.0 (2)		1.0
Lymphatic metastasis	6.4 (14)	0 (0)	15.1 (14)		14.8 (4)	0 (0)	50.0 (4)		0.24
Common bile duct invasion	3.7 (8)	0 (0)	8.6 (8)		3.7 (1)	0(0)	12.5(1)		1.0
Peripancreatic fat invasion	10.6 (23)	0 (0)	24.7 (23)		7.4 (2)	0 (0)	25.0 (2)		1.0
Duodenum invasion	6.4 (14)	0 (0)	15.1 (14)		0 (0)	0 (0)	0 (0)		0.38
**Margin status**									
Negative	80.3 (175)	88.8 (111)	68.2 (64)	**< 0.0001**	88.9 (24)	19 (100)	62.5 (5)	**0.019**	0.41
Noninvasive component	16.5 (36)	11.2 (14)	23.7 (22)	**0.014**	0 (0)	0 (0)	0 (0)	-	**0.018**
Invasive component	3.2 (7)	0 (0)	7.5 (7)	**< 0.0001**	11.1 (3)	0 (0)	37.5 (3)	**0.19**	**0.003**

*IQR* interquartile range, *DP* distal pancreatectomy, *PD* pancreaticoduodenectomy, *TP* total pancreatectomy, *CP* central pancreatectomy, *MCN* mucinous cystic neoplasm, *IPMN* intraductal papillary mucinous neoplasm, *BD/MD/MT* branch duct/main duct/mixed type, *G/I/P/O/M* gastric/intestinal/pancreatobiliary/oncocytic/mixed type

^a^Comparison of invasive vs non-invasive IPMN

^b^Comparison of invasive vs non-invasive MCN

^c^Comparison of total IPMN vs total MCN

Demographics and clinicopathological feature in IPMNs associated with malignancy included preoperative diabetes mellitus (*P* < 0.001) and resection procedure with PDs (*P* = 0.04). In contrast, resection procedure with DPs was associated with non-invasive IPMNs (*P* = 0.006). Most of the malignant lesions were pancreaticobiliary and oncocytic epithelial type (*P* = 0.006). More positive margin status (both non-invasive component and invasive component) on pathology were observed in malignant diseases than in non-invasive diseases (*P* = 0.014 and < 0.0001).

Males were extremely uncommon in our cohort, and all MCN patients included were females. Among them, 10 (37%) were premenopausal and 17 (63%) were peri- (*n* = 4) or postmenopausal (*n* = 13). Six (22.2%) premenopausal women presented MCNs during or shortly after pregnancy, and 5 (29.4%) peri- or postmenopausal patients presented estrogen-based high-risk tumors. No baseline demographics and pathological findings evaluated in this study were associated with the pathological finding of malignant diseases. However, the cyst size identified by pathological specimen in malignant cases was larger (median 58.5 mm) than that in non-invasive cases (40 mm) in spite of no statistical significance.

The cases of IPMN and MCN were further compared. MCNs were more common in females (*P* < 0.0001), and the patients tended to be younger than IPMNs (*P* < 0.0001). For resection procedures, DPs were more selected in MCN cases (*P* < 0.0001), and PDs were more often selected in IPMN cases (*P* < 0.0001). The peripheral invasion status was comparable in MCN and IPMN cases. However, margin involvement was significantly more in IPMN cases than that in MCN cases, with either non-invasive component (*P* = 0.018) or invasive component (*P* = 0.003).

### Recurrence data

The median follow-up time of the overall cohort was 34 months (interquartile range (IQR), 19–61 months). In total, 37 (15.1%) patients experienced postoperative disease recurrence, and 7 (2.9%) patients died (5 cases died of recurrence) during the follow-up period. Among the 37 cases of recurrence, 25 cases developed locoregional recurrence, and 12 cases developed extra-pancreatic recurrence. The median time of recurrence was 8 months (IQR, 5–13.3 months), and the median time of death was 46 months (IQR, 5.5–83 months). Moreover, 36 (97.3%) of the 37 recurrence cases developed within 2 years. The OS at 1, 3, and 5 years of MCNs and IPMNs combined after surgical resection was 98%, 95.9%, and 93%, and the DFS was 86.4%, 81.8%, and 81.3%, respectively.

For IPMNs, 34 patients (15.6%) developed postoperative recurrence, and 6 patients died (4 cases died of recurrence). Of the 93 cases of malignant IPMNs, 22 cases (23.7%) relapsed and 12 cases (9.6%) of 125 non-invasive IPMNs relapsed. The recurrence rate was significantly higher in malignant cases than in non-invasive cases (*P* = 0.005). The OS at 1, 3, and 5 years of IPMNs was 98.75%, 98.75%, and 97.5%, and the DFS of IPMNs at 1, 3, and 5 years was 85.7%, 81.1%, and 80.6%, respectively.

**Fig. 1:**
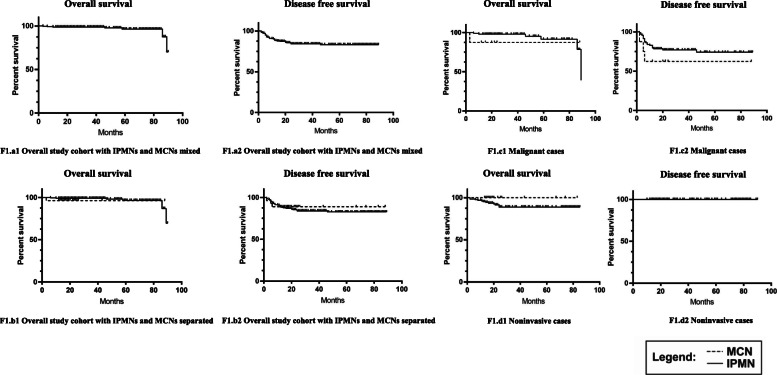
Kaplan-Meier survival curves depicting survival among MCN (dashed) and IPMN (solid). **a** Overall study cohort with IPMNs and MCNs cases mixed. **b** Overall study cohort with IPMNs and MCNs separated. **c** Malignant IPMNs and MCNs. **d** Noninvasive IPMNs and MCNs

For MCNs, 3 malignant cases of the 27 patients (11.1%) developed postoperative recurrence. Only 1 malignant case died after surgical resection due to the extra-pancreatic recurrence (omentum majus). No non-invasive cases relapsed or died during the follow-up time. Similarly, the recurrence rate was higher in malignant cases than in non-invasive cases (*P* = 0.019). The OS at 1, 3, and 5 years of MCNs was 95.7%, 95.7%, and 95.7%, and the DFS of MCNs at 1, 3, and 5 years was 91.3%, 87.0%, and 87.0%, respectively. Detailed recurrence data are presented in Table 2 with corresponding Kaplan-Meier curves in Fig. 1.

**Table 2:** Summary of results of Kaplan-Meier survival analyses

	Full cohort	Full cohort		Malignant		Noninvasive	
	Total	IPMN	MCN	IPMN	MCN	IPMN	MCN
**Overall survival**							
At risk, (*n*)	245	218	27	93	8	125	19
Median time, months (IQR)	34 (19–61)	38.5 (20–63)	24 (16–25)	35 (19–63)	20 (13–23.5)	42 (20–64)	20 (16–26)
Deaths, % (*n*)	2.9% (7)	2.8% (6)	3.7% (1)	6.5% (6)	12.5% (1)	0% (0)	0% (0)
1-year, %	98%	98.75%	95.7%	97.1%	87.5%	100%	100%
3-year, %	95.9%	98.75%	95.7%	94.2%	87.5%	100%	100%
5-year, %	93%	97.5%	95.7%	91.3%	87.5%	100%	100%
Log-rank, (*p*)		*p* = 0.48		*p* = 0.33		*p* = 1.0	
**Disease-free survival**							
At risk, (*n*)	245	218	27	93	8	125	19
Median time, months (IQR)	24 (16–53)	26 (17–54)	20 (14–25)	23 (14–52)	18 (8–24)	30 (19–58)	20 (16–26)
Recurrence, % (*n*)	15.1% (37)	15.6% (34)	11.1% (3)	23.7% (22)	37.5% (3)	9.6% (12)	0% (0)
1-year, %	86.4%	85.7%	91.3%	75.3%	75.0%	94%	100%
3-year, %	81.8%	81.1%	87.0%	74.0%	62.5%	90%	100%
5-year, %	81.3%	80.6%	87.0%	71.4%	62.5%	88%	100%
Log-rank, (*p*)		*p* = 0.67		*p* = 0.27		*p* = 0.2	

*IQR* interquartile range, *p p* value, *MCN* mucinous cystic neoplasm, *IPMN* intraductal papillary mucinous neoplasm

According to Kaplan-Meier survival analyses, MCNs and IPMNs had comparable OS and DFS after surgery in overall cohort (*P* = 0.48 and 0.67), malignant cases (*P* = 0.33 and 0.27), and non-invasive cases (*P* = 1.0 and 0.2).

### Factors associated with recurrence

Only 3 patients with MCNs relapsed, so to build a logistic regression model for MCN patients alone was lack of significance. Therefore, we established the logistic regression model of the overall study cohort. In addition, due to the small number of cases in each group, we combined the peripheral invasion status (perineural invasion, vascular invasion, cancerization of ducts, lymphatic metastasis, common bile duct invasion, peripancreatic fat invasion, and duodenum invasion) together, and in total, 37 patients had peripheral invasions. Univariate analysis showed that pathological diagnoses with malignancy (*P* < 0.0001), presence of oncocytic type for IPMN (*P* = 0.01), resection procedure with PD (*P* = 0.013), peripheral invasion (*P* < 0.0001), and incisal margin invasion (*P* = 0.02) were significant pathological risk factors for recurrence. Furthermore, we found four independent risk factors in multivariate analysis: pathological diagnoses with malignancy (*P* < 0.0001, odds ratio (OR) = 3.65), presence of oncocytic type for IPMN (*P* = 0.031, OR = 1.69), peripheral invasion (*P* < 0.0001, OR = 12.87), and incisal margin invasion (*P* = 0.039, OR = 1.99) (Table 3).

**Table 3:** Risk factors for development of recurrence tumor after surgical resection of IPMN and MCN on the univariate and multivariate analyses (*n* = 245)

	Univariate analysis			Multivariate analysis		
	Recurrence (+) *n* = 37	Recurrence (−) *n* = 208	*p* value	*p*	Odds ratio (OR)	95% confidence interval
**Age**, median years (IQR)	64 (54.5–71)	63 (54–70)	0.43			
**Sex**, male, % (*n*)	59.5 (22)	61.1 (127)	0.85			
**Diabetes mellitus**, % (*n*)	32.4 (12)	19.7 (41)	**0.08**	0.32		
**Pathological diagnoses**						
Malignant, % (*n*)	67.6 (25)	32.7 (68)	**< 0.0001**	**< 0.0001**	3.65	2.2–6.5
**Tumor size**, median mm (IQR)	50 (30–70)	40 (24–70)	0.49			
**Morphological type for IPMN,** ***n*** **= 218, recurrence (*n*** **= 34)**						
Branch duct type, % (*n*)	17.6 (6)	15.2 (28)	0.72			
**Epithelial subtype for IPMN,** ***n*** **= 218, recurrence (*n*** **= 34)**						
Gastric type, % (*n*)	32.4 (11)	43.5 (80)	0.41			
Intestinal type, % (*n*)	35.3 (12)	37.5 (69)	0.81			
Pancreatobiliary type, % (*n*)	11.8 (4)	12.0 (22)	0.98			
Oncocytic type, % (*n*)	14.7 (5)	2.2 (4)	**0.01**	**0.031**	1.69	1.13–1.97
Mixed type, % (*n*)	5.9 (2)	4.9 (9)	1.0			
**Resection procedure**						
TP, % (*n*)	5.4 (2)	6.3 (13)	1.0			
PD, % (*n*)	75.7 (28)	53.8 (112)	**0.013**	0.12		
DP, % (*n*)	16.2 (6)	12.5 (26)	0.54			
CP, % (*n*)	2.7 (1)	7.7 (16)	0.45			
Enucleation, % (*n*)	0 (0)	1.9 (4)	1.0			
**Peripheral invasion**, % (*n*)	73.0 (27)	4.8 (10)	**< 0.0001**	**< 0.0001**	12.87	1.87–24.03
**Margin status**						
Margin with noninvasive component, % (*n*)	13.5 (5)	14.9 (31)	0.82			
Margin with invasive component, % (*n*)	13.5 (5)	2.4 (5)	**0.02**	**0.039**	1.99	1.08–3.06

*IPMN* intraductal papillary mucinous neoplasm, *IQR* interquartile range, *DP* distal pancreatectomy, *PD* pancreaticoduodenectomy, *TP* total pancreatectomy, *CP* central pancreatectomy

## Discussion

This study was conducted in a high-volume pancreatic center with a large cohort. MCNs and IPMNs have the potential of malignant transformation, which is the cause of concern. Surgical resection of them with high-grade dysplasia or invasive cancer has been widely accepted. To determine the optimal high-risk patients for surgical resection, the preoperative imaging risk factor evaluation was recommended by current guidelines [13–15, 17], and the evidence level of imaging evaluation was high. Therefore, imaging parameters were not included in our study in order to avoid repetitive work. We focused on the postoperative pathological features, recurrence rate, and recurrence factors of IPMNs and MCNs. Although many studies have discussed the parameters in IPMN [18–21], there are few studies on these issues of MCNs.

The prognosis is favorable for IPMN patients after surgery. A recent study with large cohort conducted by Griffin et al. concluded that IPMN patients after surgery had a 1-year survival rate of 92.7%, and the 5-year survival rate was 72.9% [22]. Another study by Min et al. concluded that the 5-year disease-specific survival of IPMN was 83.7–100% and the 5-year recurrence-free survival was 73.1–95% [23]. A multicenter study of 1074 resected IPMNs in Japan concluded that 14.4% of patients relapsed [18]. In our study, the 1-year and 5-year OS for IPMN were 98.75% and 97.5%, and the DFS rate was 85.7% and 80.6%, respectively. The data in our study were close to the reported data. However, the 5-year OS decreased to 36.0–57.7% as reported by Griffin et al. [22] for invasive IPMNs, but the data in our study was 91.3%. The bias may be due to the exclusion criteria. In our study, perioperative deaths were excluded. We deemed that these deaths may not reflect the real harm of the diseases. Moreover, the patients in our study were younger (median, 63 years vs 69 years), and the survival curve in our study (Fig. 1) showed an obvious downward trend with the extension of follow-up time. Therefore, the data in our study may not be significantly different from other studies, and prospective studies with the same exclusion criteria are needed to address the problems of real OS and DFS of IPMNs.

MCNs accounted for nearly one fifth of all pancreatic cystic neoplasms. Unlike the more common IPMNs, of which the International Consensus Guidelines in 2006 and 2012 have offered clear criteria for surveillance or resection, surgical resection was recommended for all MCN patients considered candidates for surgery in both guidelines [10, 11]. Since then, however, many studies have opposed the aggressive recommendation because MCN is recognized as an indolent tumor. Griffin et al. concluded that MCN had a better prognosis compared to IPMN, and the postoperative 10-year survival rate approached 80% even in invasive diseases [22]. Postlewait et al. concluded that MCN patients with adenocarcinoma had a 3-year OS and DFS of 59% and 64%, respectively [24]. In a multi-institutional, retrospective study conducted by Yamao et al., the 3-, 5-, and 10-year survival rates were 97.6%, 96.6%, and 96.6%, respectively [25]. In our study, only 1 in 8 malignant MCN patients died due to the diseases, and the OS and DFS were very similar to the reported data. In summary, MCN has a favorable prognosis even in malignant cases. Therefore, a more conservative attitude towards surgical resection of MCNs should be adopted.

The characteristic subepithelial tissue of MCN is ovarian-type stroma. MCN occurs almost exclusively in middle-aged women, and some studies have linked it to pregnancy and hormone-replacement therapy [26–28]. In our study, only 6 patients (22.2%) developed MCN during or shortly after pregnancy, but it was enough to conclude that MCN may be hormonally influenced. The role of hormones in the formation and malignant transformation of MCN needs to be further studied, which may affect the management of MCN in pregnant and postmenopausal patients.

Many factors have been reported to be associated with tumor recurrence (e.g., symptoms main duct size ≥ 10 mm, mural nodule size ≥ 5 mm, presence of invasive cancer, lymph node metastasis) [18, 19]. The recurrence factors identified in our study were pathological diagnoses with malignancy, presence of oncocytic type for IPMN, peripheral invasion, and incisal margin invasion. Although these factors seemed to be diverse, they have one trait in common: they were predictors or characteristic of malignant cases. In other words, malignant cases tended to recur. We should adopt a more active surveillance strategy for those with a malignant pathological-related diagnosis no matter in IPMN or MCN cases. A timely treatment for the recurrent tumor could result in a better prognosis.

However, there was little data to guide the optimal surveillance interval after surgical resection. The latest 2018 American Committee of Gastroenterology guidelines recommended that the surveillance interval should be 6 months for IPMN patients [15]. The surveillance interval of malignant MCNs should follow the guidelines for pancreatic cancer. For benign MCNs, no further surveillance was required [15]. In our study, the median time of recurrence was 8 months (IQR, 5–13.3 months), and recurrence was more common in malignant cases. The data in our study was very similar to the recommendations in the guidelines. Therefore, we believed that it was reasonable to use 6 months as the surveillance interval. In other words, patients with high-risk recurrence factors (diagnosed with malignancy, presence of oncocytic type for IPMN, peripheral invasion, and incisal margin invasion) should undergo regular surveillance every 6 months. The optimal surveillance modalities should be MRI/CT/EUS as we adopted in our study and the guidelines recommended [15].

Our study had several limitations worth discussing. First, it was a retrospective study. The patients were often transferred from other referral institutions. Their initial medical records were not fully presented in our medical system. These patients usually transitioned care closer to the originating hospital following surgery, which also decreased the availability and completeness of long-term and follow-up data collection. The second limitation was the rarity of MCNs, which greatly limited the size of a single institution series. When we designed the study, we tried to build a logistic regression model for MCN to determine which factors were associated with recurrence. However, only 3 MCN patients showed recurrence, which limited the statistical significance of the model.

## Conclusions

This is a large single-institution series of IPMNs and MCNs reported to date with 245 cases confirmed by surgical pathology review. Based on our results, IPMNs and MCNs had comparable and favorable prognoses after surgical resection. Recurrence occurred in 14.9% of all the cases. The 5-year OS and DFS for IPMNs approached over 91% and 71% even in malignant cases, and the 5-year OS and DFS for malignant MCN cases approached over 87% and 72%. For those cases without malignancy, OS and DFS approached nearly 100%. In logistic regression analyses, pathological diagnoses with malignancy, presence of oncocytic type for IPMN, peripheral invasion, and incisal margin invasion were independently associated with tumor recurrence. The results may be beneficial to the identification of IPMN and MCN patients with high risk of postoperative recurrence and further surveillance.

## Data Availability

Please contact the corresponding authors for data requests.

## Abbreviations

PCN: Cystic neoplasms of the pancreas
IPMN: Intraductal papillary mucinous neoplasm
MCN: Mucinous cystic neoplasm
BD: Branch duct
MD: Main duct
MT: Mixed type
CT: Computed tomography
MRI: Magnetic resonance imaging
EUS: Endoscopic ultrasound
CEA: Carcinoembryonic antigen
FNA: Fine needle aspiration
DP: Distal pancreatectomy
PD: Pancreaticoduodenectomy
TP: Total pancreatectomy
CP: Central pancreatectomy
LGD: Low grade of dysplasia
IGD: Intermediate grade of dysplasia
HGD: High grade of dysplasia
OS: Overall survival
DFS: Disease free survival
